# *Campylobacter jejuni* from Canine and Bovine Cases of Campylobacteriosis Express High Antimicrobial Resistance Rates against (Fluoro)quinolones and Tetracyclines

**DOI:** 10.3390/pathogens9090691

**Published:** 2020-08-23

**Authors:** Sarah Moser, Helena Seth-Smith, Adrian Egli, Sonja Kittl, Gudrun Overesch

**Affiliations:** 1 Institute of Veterinary Bacteriology, University of Bern, 3001 Bern, Switzerland; sarah.moser@vetsuisse.unibe.ch (S.M.); sonja.kittl@vetsuisse.unibe.ch (S.K.); 2 Applied Microbiology Research, Department of Biomedicine, University of Basel, 4001 Basel, Switzerland; Helena.Seth-Smith@usb.ch (H.S.-S.); adrian.egli@usb.ch (A.E.); 3 Division of Clinical Bacteriology and Mycology, University Hospital Basel, 4001 Basel, Switzerland

**Keywords:** *Campylobacter coli*, resistance genes, one health, critically important antimicrobials

## Abstract

*Campylobacter* (*C.*) spp. from poultry is the main source of foodborne human campylobacteriosis, but diseased pets and cattle shedding *Campylobacter* spp. may contribute sporadically as a source of human infection. As fluoroquinolones are one of the drugs of choice for the treatment of severe human campylobacteriosis, the resistance rates of *C. jejuni* and *C. coli* from poultry against antibiotics, including fluoroquinolones, are monitored within the European program on antimicrobial resistance (AMR) in livestock. However, much less is published on the AMR rates of *C.* *jejuni* and *C. coli* from pets and cattle. Therefore, *C. jejuni* and *C. coli* isolated from diseased animals were tested phenotypically for AMR, and associated AMR genes or mutations were identified by whole genome sequencing. High rates of resistance to (fluoro)quinolones (41%) and tetracyclines (61.1%) were found in *C. jejuni* (n = 29/66). (Fluoro)quinolone resistance was associated with the known point mutation in the quinolone resistance-determining region (QRDR) of *gyrA*, and tetracycline resistance was mostly caused by the *tet(O)* gene. These high rates of resistance, especially to critically important antibiotics in *C. jejuni* and *C. coli*, are worrisome not only in veterinary medicine. Efforts to preserve the efficacy of important antimicrobial treatment options in human and veterinary medicine have to be strengthened in the future.

## 1. Introduction

Human campylobacteriosis is the most common cause of bacterial gastroenteritis worldwide. *Campylobacter* (*C.*) *jejuni* and *C. coli* are the most frequently isolated species in humans with diarrhea [1]. In most cases, the infection is foodborne, from handling or eating undercooked poultry, raw milk, or contaminated water. In Europe, more than 240,000 cases were reported in 2018 [1]. Source attribution studies identified *C. jejuni* and *C. coli* from poultry as the main source of human campylobacteriosis [2]. Therefore, antimicrobial resistance (AMR) in *C. jejuni* and *C. coli* is a key element of European AMR monitoring. High to very high rates of resistance of *Campylobacter* spp. from poultry resistant to antimicrobials that are critically important for humans, such as (fluoro)quinolones, have been detected with an increasing trend over the last 10 years [3]. The results from a Swiss AMR monitoring program using cecum samples from broilers showed that 51.4% of *C. jejuni* were resistant to fluoroquinolones and 40.0% to tetracycline. Moreover, 66.7% of *C. coli* in these broilers were resistant to fluoroquinolones and 40.0% to tetracycline. A similar situation exists for porcine *Campylobacter* spp. About half of the tested isolates were resistant to quinolones (50.3% to ciprofloxacin and 52.2% to nalidixic acid). Furthermore, 62.1% of these strains were resistant to tetracycline [3].

On the other hand, much less is known about the *Campylobacter* spp. strains isolated from clinical cases of cattle and pets. Although not identified as a main source of human campylobacteriosis, *Campylobacter* spp. from these animal species could contribute to human cases, and transmission to humans is possible. Campylobacteriosis in pets and cattle occurs only sporadically, and it is not recommended to treat infected animals even with symptoms like diarrhea with antibiotics, unless there is a risk of sepsis [4,5]. Therefore, data on AMR for these pathogens are rare. In studies analyzing antimicrobial susceptibility to ciprofloxacin of *C. jejuni* from dogs, major differences depending on the country could be observed [6,7,8,9,10]. It must be considered that different methods of antimicrobial susceptibility testing (AST; E-test, agar dilution, disk diffusion, microbroth dilution, or multilocus sequence typing) and different breakpoints and cut-off values are used. 

In *Campylobacter*, point mutations in the quinolone resistance-determining region (QRDR) of *gyrA* are most often responsible for resistance to fluoroquinolones [11]. Specific single point mutations in the QRDR can reduce the susceptibility of *Campylobacter* spp. to fluoroquinolones considerably [12], while avoiding fitness costs [13]. The resistance of *Campylobacter* spp. to tetracycline is normally mediated by the acquisition of the *tet(O)* gene, which can be located on the chromosome or on transferable plasmids [14,15]. In a study from Italy with *C. coli* from pigs, they found a strong correlation between phenotypic and genotypic resistance to fluoroquinolones, on the one hand, and tetracycline on the other. In particular, point mutations in *gyrA* and the presence of *tet(O)* were associated with such resistance [16].

The aim of this study was to determine the AMR rates of *C. jejuni* and *C. coli* isolated from diseased dogs, cats, and cattle for critically important antimicrobials using the European-wide harmonized method, and to compare these data to the known resistance rates of regularly monitored livestock animals. Furthermore, the molecular mechanisms responsible for phenotypic AMR were analyzed using whole genome sequencing.

## 2. Results

### 2.1. Phenotypic Microbiological Resistance

In canine *C. jejuni* (n = 39), the rates of microbiological resistance against (fluoro)quinolones were highest (ciprofloxacin (CIP): 38.5% (95% CI: 24.9–54.1); nalidixic acid (NAL) 41.0% (95% CI: 27.1–56.6)), followed by resistance against tetracycline (TET) (28.2% (95% CI: 12.7–38.3 95% CI)) (Table 1). The difference between NAL and CIP is due to one isolate resistant to NAL but not to CIP even after retesting. Resistance against streptomycin (STR) was found in 5.1% (95% CI: 11.4–16.9) of isolates, whereas no resistance against erythromycin (ERY) or gentamicin (GEN) was detected. The same pattern was found for bovine *C. jejuni* isolates (n = 18). The highest resistance rates were measured against NAL and CIP (61.1% (95% CI: 38.6–79.7)), followed by TET (33.3% (95% CI: 16.3–56.3)) and STR (5.6% (95% CI: 1.0–25.8)). Again, no resistance against ERY or GEN was detected. In cats, resistance to (fluoro)quinolones was found in three of nine isolates of *C. jejuni*.

One canine *C. coli* isolate was resistant to CIP, NAL, and STR. All eight tested isolates of bovine *C. coli* showed resistance to STR. Six of the eight tested bovine *C. coli* isolates were resistant to both (fluoro)quinolones and TET. Five isolates showed an additional resistance to ERY. No *C. coli* isolates from cats were available.

Of the 75 tested *C. jejuni/coli* isolates, 25 isolates showed phenotypical microbiological resistance against only one antimicrobial class (Table 2). Eighteen isolates were only resistant to CIP-NAL, five strains only to TET, and two strains exclusively to STR. The most often found resistance pattern in *C. jejuni* was CIP-NAL (18/36), and CIP-NAL-ERY-TET-STR in bovine *C. coli* (5/9). Of the 66 tested *C. jejuni*, 13 were fully susceptible to all the antimicrobials tested, whereas none of the nine *C. coli* were fully susceptible.

Because of the limited number of isolates, the calculated confidence intervals overlap to a great extent. Therefore, further statistical analyses were not performed.

### 2.2. Antimicrobial Resistance Genes

Whole genome sequencing was performed on all the strains with phenotypic AMR (n = 45). There were 36 *C. jejuni* strains (canine n = 21, feline n = 3, bovine n = 12) and nine *C. coli* strains (canine n = 1, bovine n = 8). The phenotypic AMR pattern and the presence of corresponding AMR genes and point mutations are listed in Table 2.

Resistance to both NAL and CIP (n = 36) was found to be caused by a point mutation resulting in T86I in the QRDR of *gyrA*. This mutation was not found in the one *C. jejuni* isolate resistant to NAL but not to CIP. Three TET resistance ribosomal protection proteins were identified in isolates showing non-wild-type TET minimum inhibitory concentration (MIC) (n = 24). *tet(O)* was the most common gene and was detected in 16 isolates. Furthermore, the mosaic *tet(O/32/O)* was identified in two *C. jejuni* strains, and *tet(W)* was found in one bovine *C. coli* isolate. Phenotypic TET resistance in five isolates could not be assigned to a known resistance gene. Various resistance genes and one single point mutation were associated with resistance to STR. In one *C. jejuni* isolate, a point mutation in *rpsL* leading to an amino acid change K88R was detected. The *aadE-Cc* gene was discovered in four *C. coli* isolates and one *C. jejuni* isolate. Three bovine *C. coli* strains harbored the cassette *aadE-Cc*, *ant(6)-Ia, aph(3′)-IIIa, sat4.* These isolates were resistant to all tested antimicrobials, except GEN. Additionally, *cfr(C)* was present in these three isolates, which could be linked to ERY resistance. In the two other isolates resistant to ERY, the point mutation A2075G in the 23S rRNA gene was observed.

## 3. Discussion

In our study, AMR to (fluoro)quinolones was slightly higher in canine *C. jejuni* isolates (n = 15/39; 38.5% (95% CI: 24.9–54.1) than in a study from 2014 in Switzerland (n = 28/134; 20.9% (95% CI: 14.9–28.5)), which typed the isolates with MLST and *fla*-typing [10]. This could be an indication of increasing resistance to quinolones in companion animals. In Switzerland, a statistically significant increase in the resistance of *C. jejuni* to CIP in broilers has been observed over the past 10 years [3]. Because (fluoro)quinolone resistance is not associated with a loss of fitness, a rapid development of resistance occurs [13]. The resistance rate of *C. jejuni* and *C. coli* to CIP in diseased cattle (61.1% (95% CI: 38.6–79.7) in our study is comparably high to that in slaughter pigs (50.3% (95% CI: 42.7–57.9)) and slaughter broilers (54.1% (95% CI: 46.2–61.4)), which are regularly monitored in Switzerland [3]. It is interesting, as one might expect, that the resistance rates are higher in diseased and therefore possibly treated animals than in healthy slaughter animals. Moreover, antimicrobial treatment regimens for livestock species differ markedly—e.g., for broilers in particular, only oral treatment is applied, as opposed to cattle, where other treatment routes are common. The resistance of *C. jejuni* to TET is lower in the isolates of this study than in the broilers of AMR monitoring. In contrast to the results of the AMR monitoring, no isolate of *C. jejuni* resistant to GEN or ERY was found in our study, whereas the ERY and GEN resistance of *C. jejuni* in broilers is very low but does occur [3]. The resistance of *C. jejuni* to STR is comparable to the resistance of Swiss broilers harboring *C. jejuni* as a commensal. All the tested *C. coli* were resistant to STR. In other studies, high resistance rates of *C. coli* to STR are described [3,17]. The resistance rate to STR in this study was higher compared to other aminoglycosides such as GEN, because STR binds to a single site on the 30S subunit of the ribosome and high-level resistance can be selected through a single mutation [18]. All the resistance rates in cats and all the resistance rates for *C. coli* can only be evaluated to a limited extent due to the low number of cases.

Fluoroquinolone resistance often arises from point mutations in the QRDR of *gyrA*, with the resulting amino acid change C257T being the most common [11,19]. Mutations in *gyrA* leading to T86I were found in all the isolates resistant to CIP and NAL, except for one strain, which was only resistant to NAL and not to CIP. This phenomenon was described by Jesse et al. (2006), who detected a single Thr86Ala mutation in *Campylobacter*, leading to phenotypic resistance to NAL but not to CIP [20]. However, this mutation was not detected in our strain. Three tetracycline ribosomal protection proteins were detected in our study; *tet(O)* was detected most frequently, followed by *tet(O/32/O)* and *tet(W)*. The *tet(O)* gene widely occurs in *C. coli* and *C. jejuni* and can be located either on the chromosome or on a plasmid [21,22,23]. The location of *tet(O)* was not analyzed in this study. The *tet(O/32/O)* gene was detected in two isolates of *C. jejuni*. Only a small amount of data on *tet(O/32/O)* has been published up to now. A British study found *tet(O/32/O)* in *C. jejuni*; this gene can also either be on plasmids or on the chromosome [24]. The locations of *tet(O)* and *tet(O/32/O)* were not determined in that study. *Tet(O/32/O)* was discovered in *Streptococcus suis* from diseased pigs in China and the fecal samples of humans from several European countries [25,26]. It is likely that *Campylobacter* spp. acquired *tet(O/32/O)* from other intestinal bacteria.

The third ribosomal protection protein, *tet(W)*, was detected in a bovine isolate of *C. coli*. Previous studies found *tet(W)* widely distributed in the genome of ruminal bacteria of cattle, and the authors assumed that antimicrobial treatment is the driver of positive selection for tetracycline-resistant bacteria [27]. Holman et al. found that the use of oxytetracycline increased the proportion of *tet(W)* in fecal samples [28]. These studies show that the *tet(W)* gene can be present in the microbiome of the digestive tract and can be transferred to other bacteria.

All five ERY-resistant *C. coli* isolates showed very high MICs (>128 mg/L) and were isolated from cattle. For two of these resistant strains, the causal mutation is in domain V of the 23S rRNA gene and occurs at position 2075, which leads to a substitution from A to G. This point mutation is the predominant mutation in clinical and field isolates [11,19]. Probably, the cause of ERY resistance in the other three strains is the presence of *cfr(C)*, which could only be detected in these isolates. *Cfr(C)* is an rRNA methyltransferase that is normally linked with resistance to phenicols, lincosamides, oxazolidines, pleuromutilins, and streptogramin A [29,30]. A Chinese study showed that *cfr(C)*-harboring *C. coli* showed an increased MIC to erythromycin, but not to phenicols. The authors suggested that *cfr(C)* was suppressed by unknown mechanisms. Moreover, they observed that *cfr(C)* was located next to the gene cluster *aadE-aphA3-sat4* [31]. The three strains in our study carrying *cfr(C)* also harbored the gene cluster and were multidrug-resistant to CIP, NAL, TET, ERY, and STR. Resistance to STR can be caused by several genes and mutations. A mutation causing K88R in *rpsL* was detected in one of our bovine *C. jejuni* isolates; that this causes a high MIC with STR in *Campylobacter* spp. has been confirmed in other studies [32]. The most frequently discovered acquired resistance gene in STR-resistant isolates in our study was *aadE-Cc,* either alone or as cassette with *ant(6)-Ia, aph(3*′)-*IIIa*, and *sat4*. The most prevalent mechanism of aminoglycoside resistance in *Campylobacter* spp. and other bacteria is the modification of the aminoglycoside structure by enzymes such as aminoglycoside acetyltransferases, aminoglycoside phosphotransferases, and aminoglycoside nucleotidyltransferases [33,34]. The presence of the *aadE-aphA-3-sat4* cluster was observed earlier in both *C. jejuni* and *C. coli* in several publications [35,36]. In contrast to other studies, no resistance to GEN was found in our isolates [37].

In general, a strong correlation of detected AMR genes and point mutations and phenotypic resistance could be observed for all the groups of antimicrobials. In three *C. jejuni* strains and one *C. coli* strain, phenotypic resistance against TET was observed, but no corresponding gene was found. For two phenotypically STR-resistant *C. coli*, no responsible mutation or AMR gene could be confirmed. The overall low number of isolates from clinical cases of canine, feline, and bovine camplyobacteriosis limits our findings to some extent, and hampered statistical analysis in general. Hence, the results are presented solely descriptively.

## 4. Materials and Methods

Between 2015 and 2018, a total of 75 *C. jejuni/coli* strains isolated from canine, feline, and bovine clinical cases by 6 diagnostic laboratories were sent to the Swiss national reference laboratory for species identification (Table 3). There were 9 strains of *C. coli* (cattle n = 8, dog n = 1) and 66 strains of *C. jejuni* (dog n = 39, cat n = 9, cattle n = 18). Species identification was performed using matrix-assisted laser desorption/ionization time-of-flight mass spectroscopy (MALDI-TOF MS) (Biotyper 3.0, Bruker Daltonics, Bremen, Germany). Isolates were stored at –80 °C in tryptone soy medium containing 30% glycerol until AST was performed. The strains were recovered on trypticase soy agar plates with 5% sheep blood (TSA SB; Becton Dickinson, Franklin Lakes, NJ, USA) and incubated microaerobically at 37 ± 1 °C for 48 h.

AST was performed by microbroth dilution according to the guidelines of the Clinical and Laboratory Standards Institute (CLSI) against the following antimicrobials: erythromycin (ERY), ciprofloxacin (CIP), tetracycline (TET), gentamicin (GEN), nalidixic acid (NAL), and streptomycin (STR). For a standardized concentration of 5 × 10^5^ CFU/mL, a McFarland of 0.5 (Densicheck, BioMérieux, Marcy-l’Etoile, France) was set and 50 μL of this suspension was transferred to 10 mL of cation-adjusted Mueller–Hinton broth with 5% lysed horse blood (TREK Diagnostic Systems, Thermo Fisher Scientific, UK). In each well of the plate, EUCAMP2 100 μL (TREK Diagnostic Systems) was inoculated using an auto-inoculator (Thermo Fisher Scientific). The plates were incubated in a 36 ± 1 °C microaerobic atmosphere for 48 h. The minimal inhibitory concentration (MIC) was defined as the lowest concentration of antibiotic showing no growth. The isolates were defined as microbiologically susceptible or resistant according to the epidemiological cut-off values (ECOFFs) issued by the European Committee on Antimicrobial Susceptibility Testing (www.eucast.org; MIC and zone diameter distributions and ECOFFs, version 5.26).

Whole genome sequencing was performed on isolates after DNA extraction on a Qiagen Qiacube using the QIAamp DNA mini kit, with Nextera XT or Nexteralexlibrary production and sequencing at 24- to 48-plex on a MiSeq with 2 × 300 bp or 96-plex on a NextSeq 500 with a 2 × 150 bp to minimum 40× mean coverage. Assemblies were performed using Unicycler v0.3.0b [38]. All the raw data have been deposited with the European Nucelotide Archive (ENA) under project number PRJEB39858.

The identification of determinants to quinolones, erythromycin, aminoglycosides, and tetracycline was performed with ABRicate (https://github.com/tseemann/abricate) using the National Center for Biotechnology Information (NCBI) database [39], ResFinder 3.0 [40], and CARD [41], with a threshold for the identification of acquired genes of 90% identity and 60% minimum length. ABRicate does not detect mutations, hence ResFinder 3.0 and CARD were used to detect point mutations. ResFinder 3.0 did not find point mutations in *C. coli*.

## 5. Conclusions

The known high resistance rate of *C. jejuni* to critically important (fluoro)quinolones in broilers in Europe is also seen in Swiss *C. jejuni* isolated from bovine campylobacteriosis cases (61.1%) and, to a lesser extent, in canine campylobacteriosis cases (38.5%). This (fluoro)quinolone resistance is caused by a known point mutation in the QRDR of *gyrA*. Moreover, tetracycline resistance as a result of the presence of *tet(O)*, *tet(W),* and *tet(O/32/O)* was observed in bovine (33.3%) and canine (23.1%) *C. jejuni* isolates. In general, the number of *C. coli* isolates resistant to at least one antimicrobial tested was high, but it has to be considered that the number of isolates was very low. The finding of high resistance rates of *Campylobacter* spp. to critically important antimicrobials in our study emphasizes the need for regular AMR monitoring not only in zoonotic and commensal bacteria from healthy slaughter animals, but also in pathogens from diseased livestock and companion animals.

## Figures and Tables

**Table 1:** Microbiological resistance rates of *C. jejuni* and *C. coli* isolated from canine, feline, and bovine clinical cases.

		Host		
Antimicrobial	*Campylobacter* Species	Dog (%) [95% CI]	Cat (%) [95% CI]	Cattle (%) [95% CI]
**Ciprofloxacin (CIP)**	*C. jejuni*	15/39 * (38.5%) [24.9–54.1]	3/9	11/18 (61.1%) [38.6–79.7]
	*C. coli*	1/1	0/0	6/8
**Nalidixic acid (NAL)**	*C. jejuni*	16/39 (41.0%) [27.1–56.6]	3/9	11/18 (61.1%) [38.6–79.7]
	*C. coli*	1/1	0/0	6/8
**Tetracycline (TET)**	*C. jejuni*	9/39 (23.1%) [12.7–38.3]	1/9	6/18 (33.3%) [16.3–56.3]
	*C. coli*	0/1	0/0	6/8
**Streptomycin (STR)**	*C. jejuni*	2/39 (5.1%) [11.4–16.9]	0/9	1/18 (5.6%) [1.0–25.8]
	*C. coli*	1/1	0/0	8/8
**Erythromycin (ERY)**	*C. jejuni*	0/39 (0%) [0.0–9.0]	0/9	0/18 (0%) [0.0–17.6]
	*C. coli*	0/1	0/0	5/8
**Gentamicin (GEN)**	*C. jejuni*	0/39 (0%) [0.0–9.0]	0/9	0/18 (0%) [0.0–17.6]
	*C. coli*	0/1	0/0	0/8

Note: * Number of resistant isolates/number of isolates tested (percentage of resistance). Calculations were only performed when ≥10 isolates were present. CI, confidence interval.

**Table 2:** Microbiological phenotypic resistance patterns of *C. jejuni* and *C. coli* and the corresponding genes or mutations detected.

			Antimicrobial Resistance Genes or Mutations								
Phenotypic Resistance Pattern	*Campylobacter* Species	Number of Isolates with Same Pattern (n)	CIP-NAL	TET			ERY		STR		
			GyrA T86I	*tet(O)*	*tet(O/32/O)*	*tet(W)*	*cfr(C)*	23S A2075G	*aadE-Cc*	RpsL K88R	*aadE-ant(6)-Ia-aph(3′)-IIIa-sat4*
CIP-NAL	*C. jejuni*	18 (dog = 10, cat = 2, cattle = 6)	18	n.d.	n.d.	n.d.	n.d.	n.d.	n.d.	n.d.	n.d.
TET	*C. jejuni*	5 (dog)	n.d.	3	n.d.	n.d.	n.d.	n.d.	n.d.	n.d.	n.d.
TET-STR	*C. jejuni*	1 (cattle)	n.d.	1	n.d.	n.d.	n.d.	n.d.	n.d.	1	n.d.
CIP-NAL-TET	*C. jejuni*	10 (dog = 4, cat = 1, cattle = 5)	10	8	1	n.d.	n.d.	n.d.	n.d.	n.d.	n.d.
NAL-TET-STR	*C. jejuni*	1 (dog)	n.d.	n.d.	n.d.	n.d.	n.d.	n.d.	n.d.	n.d.	n.d.
CIP-NAL-TET-STR	*C. jejuni*	1 (dog)	1	n.d.	1	n.d.	n.d.	n.d.	1	n.d.	n.d.
STR	*C. coli*	2 (cattle)	n.d.	n.d.	n.d.	n.d.	n.d.	n.d.	2	n.d.	n.d.
CIP-NAL-STR	*C. coli*	1 (dog)	1	n.d.	n.d.	n.d.	n.d.	n.d.	n.d.	n.d.	n.d.
CIP-NAL-TET-STR	*C. coli*	1 (cattle)	1	n.d.	n.d.	1	n.d.	n.d.	n.d.	n.d.	n.d.
CIP-NAL-TET-ERY-STR	*C. coli*	5 (cattle)	5	4	n.d.	n.d.	3	2	2	n.d.	3

CIP, ciprofloxacin; NAL, nalidixic acid; TET, tetracycline; STR, streptomycin; ERY, erythromycin; n.d., not detected.

**Table 3:** Number of isolates per year and animal species (total n = 75).

Animal Species	*Campylobacter* spp.	2015 (n = 36)	2016 (n = 16)	2017 (n = 22)	2018 (n = 1)	Total (n)
Dog (n = 40)	*C. jejuni*	25	6	8	0	39
	*C. coli*	1	0	0	0	1
Cat (n = 9)	*C. jejuni*	3	4	2	0	9
Cattle (n = 26)	*C. jejuni*	5	2	10	1	18
	*C. coli*	2	4	2	0	8
